# Different evolutionary trends form the twilight zone of the bacterial pan-genome

**DOI:** 10.1099/mgen.0.000670

**Published:** 2021-09-24

**Authors:** Gal Horesh, Alyce Taylor-Brown, Stephanie McGimpsey, Florent Lassalle, Jukka Corander, Eva Heinz, Nicholas R. Thomson

**Affiliations:** ^1^​ Parasites and Microbes, Wellcome Sanger Institute, Wellcome Genome Campus, Hinxton, Cambridgeshire, UK; ^2^​ Helsinki Institute for Information Technology HIIT, Department of Mathematics and Statistics, University of Helsinki, Helsinki, Finland; ^3^​ Department of Biostatistics, University of Oslo, Oslo, Norway; ^4^​ Departments of Vector Biology and Clinical Sciences, Liverpool School of Tropical Medicine, Liverpool, UK; ^5^​ Department of Infectious and Tropical Diseases, London School of Hygiene & Tropical Medicine, London, UK

**Keywords:** evolutionary dynamics, *E. coli*, HGT, pan-genome

## Abstract

The pan-genome is defined as the combined set of all genes in the gene pool of a species. Pan-genome analyses have been very useful in helping to understand different evolutionary dynamics of bacterial species: an open pan-genome often indicates a free-living lifestyle with metabolic versatility, while closed pan-genomes are linked to host-restricted, ecologically specialized bacteria. A detailed understanding of the species pan-genome has also been instrumental in tracking the phylodynamics of emerging drug resistance mechanisms and drug-resistant pathogens. However, current approaches to analyse a species’ pan-genome do not take the species population structure into account, nor do they account for the uneven sampling of different lineages, as is commonplace due to over-sampling of clinically relevant representatives. Here we present the application of a population structure-aware approach for classifying genes in a pan-genome based on within-species distribution. We demonstrate our approach on a collection of 7500 *Escherichia coli* genomes, one of the most-studied bacterial species and used as a model for an open pan-genome. We reveal clearly distinct groups of genes, clustered by different underlying evolutionary dynamics, and provide a more biologically informed and accurate description of the species’ pan-genome.

## Data Summary

The authors confirm all supporting data, code and protocols have been provided within the article or through supplementary data files. All analyses were performed using custom R and Python scripts, available at https://github.com/ghoresh11/twilight/tree/master/manuscript_scripts.

**Impact Statement**

Whole genome sequencing (WGS) studies over the last two decades have revealed that a substantial proportion of the combined pool of genes within a species – the pan-genome – has a heterogeneous distribution across its members. Traditionally, this gene pool has been divided into core genes, present across the majority of genomes, and accessory genes, whose presence vary across the dataset. These traditional methods do not reflect the true complexity of gene dynamics across a diverse species, nor do they account for population structure or the inherent biases in sampling and sequencing, especially since WGS has become more commonplace. To address this, we propose a novel framework that further divides the core and accessory categories into 13 subcategories to better account for differences between lineages at a finer scale than traditional methods. We use *Escherichia coli* as an exemplar species to explore how these categories can elucidate underlying evolutionary trajectories that are masked by the traditional binary approach. We show that this approach can be used to confirm many previously held assumptions, as well as identify novel predictive properties of lineages. This approach prompts us to re-think pan-genome analyses and gene distributions across other species of interest.

## Introduction

Advances in whole genome sequencing in the last two decades and the ability to sequence multiple isolates of the same species have revealed that, often, only a small fraction of genes are shared by all species members. Conversely, a substantial proportion of the combined pool of genes within a species – the pan-genome – consists of highly mobile genetic material with heterogeneous distributions across its members [1].

In a traditional pan-genome analysis, genes are divided into core genes, describing those present across the majority of the members of the species, and accessory genes, which are only present in some. The accessory genome is often further subdivided into rare and intermediate genes based on their frequency in the dataset. However, measuring gene frequencies across the whole dataset does not account for the population structure or biased sampling of the genomes in the dataset. Such simple classification can be particularly problematic when the population of interest consists of multiple deep-branching lineages that are unevenly represented in the collection. For example, if 50 % of a genome collection is represented by one lineage that was heavily over-sampled compared to other lineages, and all isolates of that lineage have a particular gene which is absent in all other lineages, this gene will simply be defined as an ‘intermediate’ gene. Based on these definitions alone, it would not be differentiated from a gene that is found in all isolates of all the other lineages, or evenly distributed across the different lineages comprising 50 % of the total isolates. Notably, ecological adaptation of a globally disseminated lineage may be driven by a large set of genes found in all isolates of that lineage, which are rare outside the lineage [2]. Hence, the biological reality requires more refined concepts when classifying genes in the pan-genomic context.

Here, we introduce a population structure-aware approach to classify the genes of a pan-genome beyond accessory and core categories, which accounts for the relative representation of the lineages in the population being studied. This refined classification allows us to better describe the pan-genome and its underlying evolutionary dynamics in organisms with complex population structures. Recent hypotheses on the evolution of the pan-genome have highlighted that different evolutionary mechanisms are required to explain the observed patterns of large open pan-genomes [3–6]. Several competing and non-exclusive hypotheses have been proposed, including the selectively neutral spread of accessory genes – including, but not limited to highly mobile selfish elements [3, 4], or indeed adaptive evolution [6]. Here we illustrate how an analysis of the patterns of within-species gene distribution informed by population structure can provide a more precise view of genes following different evolutionary trajectories. We demonstrate this on a compiled dataset of over 7500 carefully curated *Escherichia coli* genomes: one of the most well-studied bacterial species and used frequently as a model to illustrate an open pan-genome [7–9].

## Methods

### Gene classification into ‘distribution classes’

**Fig. 1.**
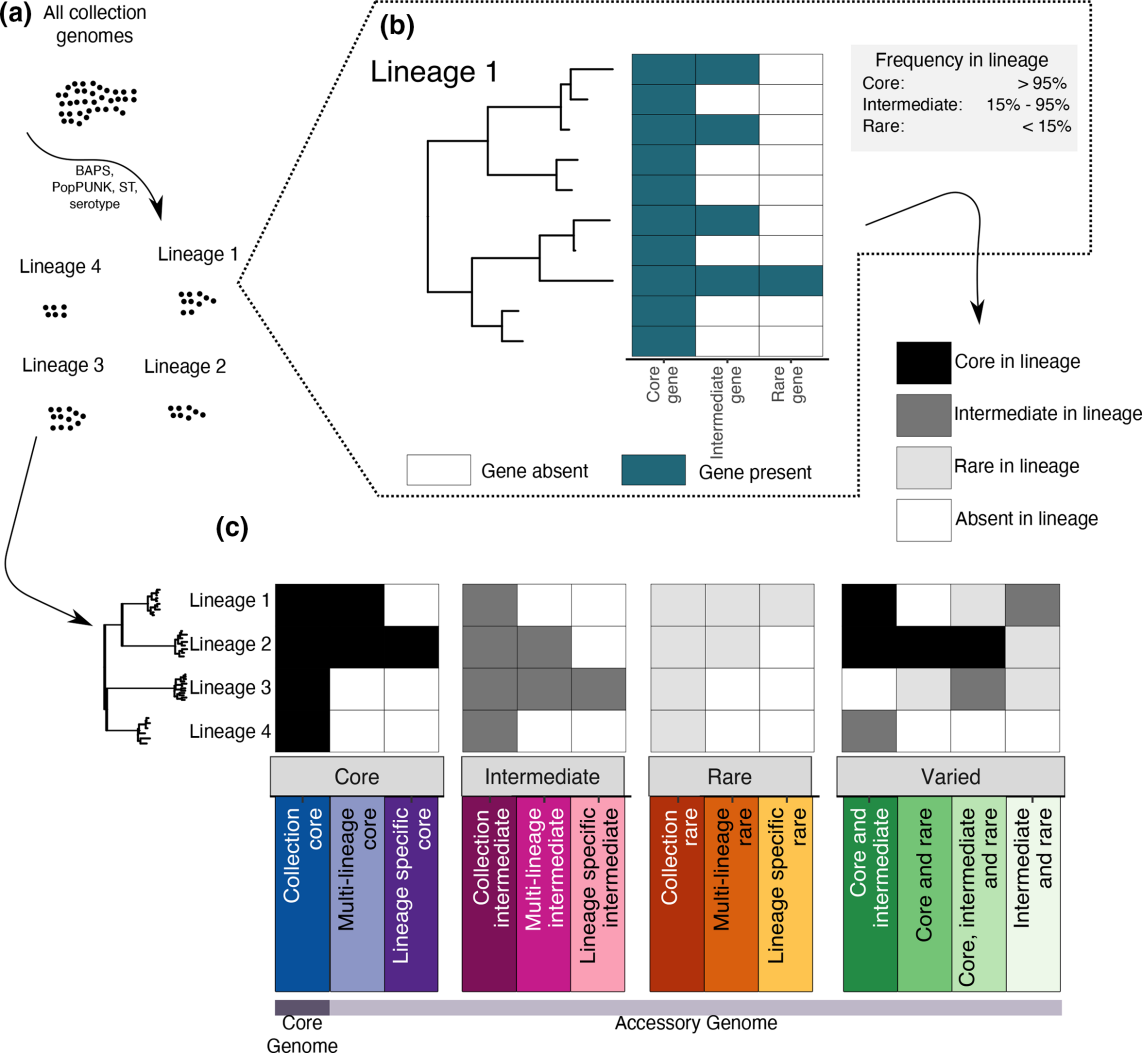
Twilight pan-genome analysis workflow. (**a)** A collection of genomes are grouped into lineages of closely related isolates. (**b)** Each gene is classified as core, intermediate or rare in each lineage, depending on its frequency within the lineage (as defined in the grey box). (**c)** The classification of the entire gene pool across all lineages consists of a total of 13 distribution classes. These include the number of lineages in which a gene is present (all lineages, multiple lineages or a single lineage), and the combination of frequency assignments of the gene in those lineages (core, intermediate or rare).

Each gene cluster was assigned to a distribution class based on its frequency within genomes belonging to the same phylogenetic clusters, termed lineages (Fig. 1a). Within each lineage, a gene was defined as ‘core’ if it was present in more than 95 % of the isolates of that lineage, ‘intermediate’ if present in 15–95 % of isolates of the lineage, and ‘rare’ if present in up to 15 % of the isolates of the lineage (Fig. 1b). Three main distribution classes, ‘Core’, ‘Intermediate’ and ‘Rare’, contained all the genes that were always observed as being ‘core’, ‘intermediate’ or ‘rare’, respectively, across the lineages in which they were present (Fig. 1c). ‘Collection core’, ‘collection intermediate’ and ‘collection rare’ genes were present and in their respective frequencies across all the lineages of the collection. ‘Multi-lineage core’, ‘multi-lineage intermediate’ and ‘multi-lineage rare’ genes were present in multiple lineages in their respective frequencies. ‘Lineage specific core’, ‘lineage specific intermediate’ and ‘lineage specific rare’ genes were present only in one lineage in their respective frequencies. The final main distribution class, or ‘varied’ genes, included all the genes which were observed as either a combination of ‘core’, ‘intermediate’ or ‘rare’ across multiple lineages. All the possible combinations are ‘core, intermediate and rare’, ‘core and intermediate’, ‘core and rare’, and ‘intermediate and rare’ (Fig. 1c). The classification of all genes in the *E. coli* collection is available in Table S1 (available in the online version of this article).

### Measuring the genetic composition of each lineage

The number of genes from each of the 13 distribution classes was counted in each of the 7693 *E. coli* genomes in the collection. The median number of genes from each distribution class was calculated per lineage. The genetic composition of a typical *E. coli* genome was measured as the median across the medians calculated per lineage for each distribution class.

### Gene-tree species-tree reconciliation

GeneRax (v1.2.2) was used to infer the probability of a horizontal gene transfer event for each gene using species-tree gene-tree reconciliation [10]. A multiple sequence alignment of all the representative sequences of each gene cluster which had at least four members (available as file F6 in Horesh *et al*. [11]) was performed using mafft (v7.310) [12]. An initial tree for each gene cluster, used as the input for GeneRax, was reconstructed using iqtree (v1.6.10) with SH-like approximate likelihood ratio test (SH-aLRT) with 1000 replicates [13] The reconciliation was performed against the species tree provided in Horesh *et al*. [11 ] with strategy SPR, reconciliation model UndatedDTL and substitution model GTR+G. The probability of transfer was inferred by GeneRax for each of the gene clusters when reconciled against the species tree.

### Counting gain events

The phylogenetic tree representing the 47 lineages was downloaded from Horesh *et al*. [11]. The phylogenetic distance between every two lineages was measured as the patristic distance using the function ‘cophenetic’ from the R package ape (v5.3) [14]. The patristic distance is the sum of the total distance between two leaves of the tree, which represent the lineages, and hence summarizes the total genetic change in the core gene alignment represented in the tree.

The leaves or tips of the phylogenetic tree represent the 47 lineages. Presence of a gene in a lineage (tree leaf) was defined as the gene being observed at least once in at least one isolate of the lineage, i.e. the frequency in the lineage was ignored. The presence or absence of a gene in an ancestral node, i.e. an internal node, was determined using accelerated transformation (ACCTRAN) reconstruction implemented in R [15]. ACCTRAN is a maximum parsimony-based approach which minimizes the number of transition events on the tree (from absence to presence and vice versa) while preferring changes along tree branches closer to the root of the tree.

Gain and loss events were counted based on the results of the ancestral state reconstruction. If there was a change from absence to presence from an ancestor to a child along a branch in the phylogeny, a gain event was counted. If there was a change from presence to absence a loss event was counted. The total number of gain and loss events was counted for each gene as well as on each branch for all distribution classes. ggtree (v1.16.6) was used for phylogenetic visualization [16].

### Measuring gene sharing between lineages

**Fig. 2.**
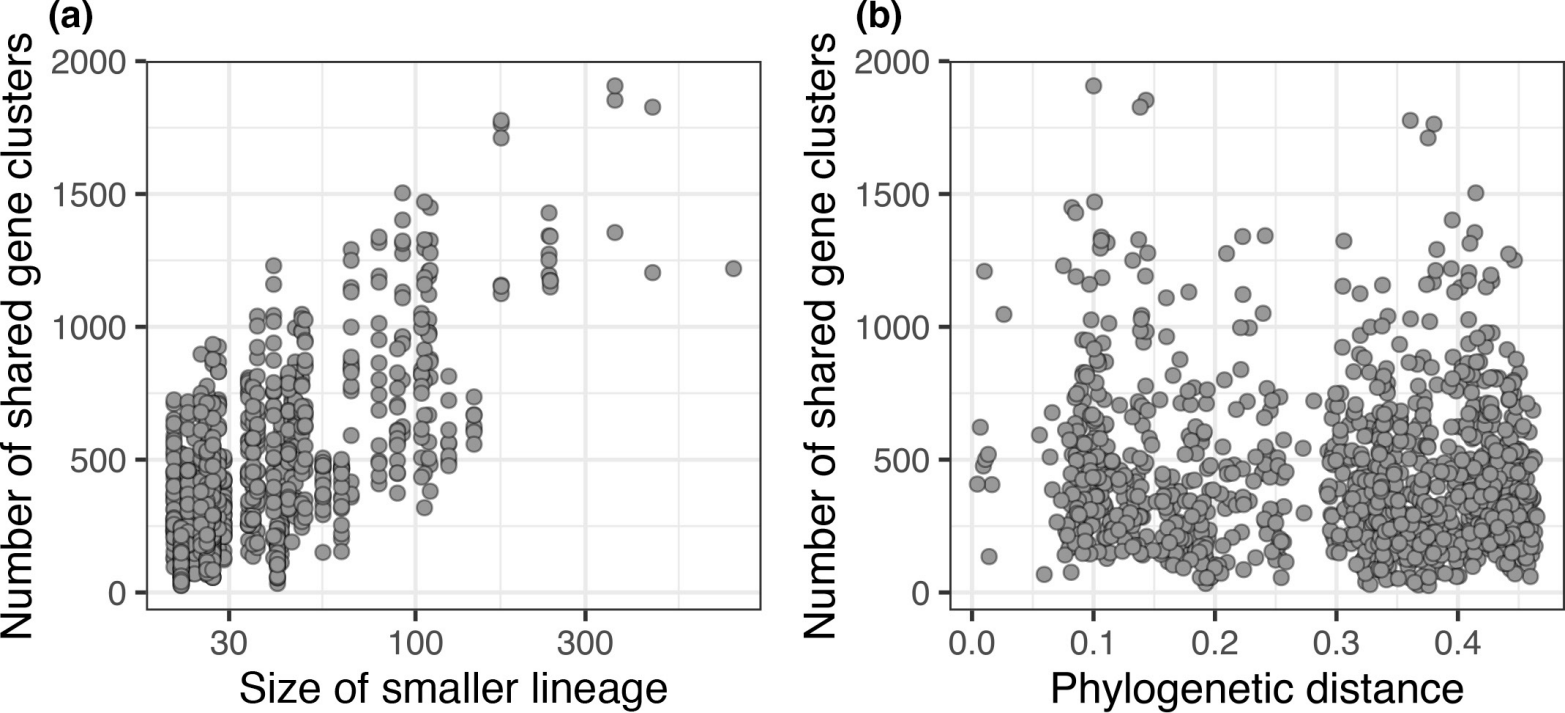
Relationship between sharing of ‘intermediate and rare’ genes, phylogenetic distance and lineage size. Relationship between the number of ‘intermediate and rare’ genes shared between every two lineages and the size of the smaller lineage of the two being compared (**a**) or the phylogenetic distance between them (**b**). Pairwise comparisons were considered between every two of the 47 lineages.

The number of genes shared from each distribution class between every two lineages was counted using custom R and Python scripts. To identify whether some lineages shared more genes than expected, we corrected for gene sharing driven by the phylogeny or by a large sample size. To correct for phylogenetically driven gene sharing, for each lineage we only counted the number of genes shared with lineages which had a patristic distance of 0.15 or more from it on the species tree. This threshold was chosen based on the observation that isolates from the same phylogroup had a patristic distance lower than 0.15 (Fig. 2). To correct for the lineage size, we subsampled each lineage to a size of 20 genomes, so that all lineages had the same size, and repeated this process 40 times. We then measured the mean number of shared ‘intermediate and rare’ genes across the 40 repeats (Fig. S1). The new counts no longer correlated with the size of the lineages (Fig. S1).

### Functional assignment of COG categories

The predicted function and COG (Clusters of Orthologous Groups) category of each gene cluster were assigned using eggNOG-mapper (1.0.3) on the representative sequence of each of the gene clusters [17]. Diamond was used for a fast local protein alignment of the representative sequences against the eggNOG protein database (implemented within eggNOG-mapper). The COG classification scheme comprises 22 COG categories which are broadly divided into functions relating to cellular processes and signalling, information storage and processing, metabolism, and genes which are poorly categorized [18]. When no match was found in the eggNOG database, the genes were marked as ‘?’ in their COG category.

Sub-sentences of all lengths were extracted from each of the functional predictions for each gene cluster using the function ‘combinations’ from the Python package ‘itertools’, while ignoring common words. For instance, for the functional prediction ‘atp-binding component of a transport system’, the words ‘of’, ‘a’ and ‘system’ were ignored, and the extracted sub-sentences were ‘atp-binding component’, ‘atp-binding component transport’ and ‘component transport’. The number of times each sub-sentence appeared in each distribution class was counted. Overlapping sub-sentences which only had a difference of 3 or smaller in their total counts per distribution class were merged in the final count to include only the longer sub-sentence. For instance, if ‘atp-binding component transport’ was counted 100 times and ‘atp-binding component’ was counted 103 times, the final count would only include the longer sub-sentence ‘atp-binding component transport’ with a count of 100.

### Code availability

The script used to classify the genes into distribution classes and generate the figures presented in this study is available at https://github.com/ghoresh11/twilight. The script can be applied on any other dataset, given a gene presence absence file as generated by pan-genome analysis tools and a grouping of each genome into a lineage. ggplot2 was used for all plotting [19].

## Results

### Case study: population structure-aware pan-genome analysis of a collection of 7500 *E. coli* genomes

To demonstrate how one can refine a pan-genome description while accounting for population structure, we used a recently published genome collection that includes over 7500 *E. coli* and *Shigella* sp. genomes isolated from human hosts, referred to as the Horesh collection [11]. Shigellae are in fact specialized pathotypes of *E. coli* and were thus included [20, 21]. Briefly, the genomes in the Horesh collection were collated from publications and other public resources, representing the known diversity of the clinical *E. coli* isolate genomes available in public databases, and underwent quality-control steps to ensure a final set of high-quality genomes. The genomes were grouped into lineages of closely related isolates (Fig. 1a) using a whole genome-based clustering method that was designed to determine bacterial within-species population structure [22]. In total, the collection featured 1158 lineages representing the *E. coli* species (as described in Horesh *et al*. [11]). We restricted our population-structure aware pan-genome analysis to the largest 47 lineages, which represented the majority of this dataset (7692/10 158 genomes). Importantly regarding the demonstration of our approach, 70 % (5349/7692) of all genomes in this collection belong to six highly overrepresented lineages, further highlighting the inherent biases that need to be overcome in such datasets. The pan-genome of the Horesh collection was classified into 50 039 homologous gene clusters (as described by Horesh [11]).

### The classical definition of the core genome is heavily influenced by the underlying biases of the studied datasets

We defined the distribution for each gene cluster in the *E. coli* and *Shigella* genome dataset by considering their frequency in each of the above-defined lineages independently. A gene cluster can thus be core, intermediate, rare or absent based on its frequency within each respective lineage (Fig. 1a, b) but can have varied distributions in different lineages (Fig. 1c, e.g. core in some and rare in other lineages). We summarized the combination of gene cluster occurrence patterns across lineages into a set of 13 species-wide distribution patterns, which we propose as novel categories for a more appropriate and complete description of datasets with complex underlying population structure (Fig. 1c). Compared to traditional pan-genome analyses, the ‘collection core’ genes represent the classical definition of the core genome, whereas we consider the accessory genome as subdivided into 12 new classes, informed by the population structure, whose distribution reflects several different evolutionary dynamics.

**Fig. 3.**
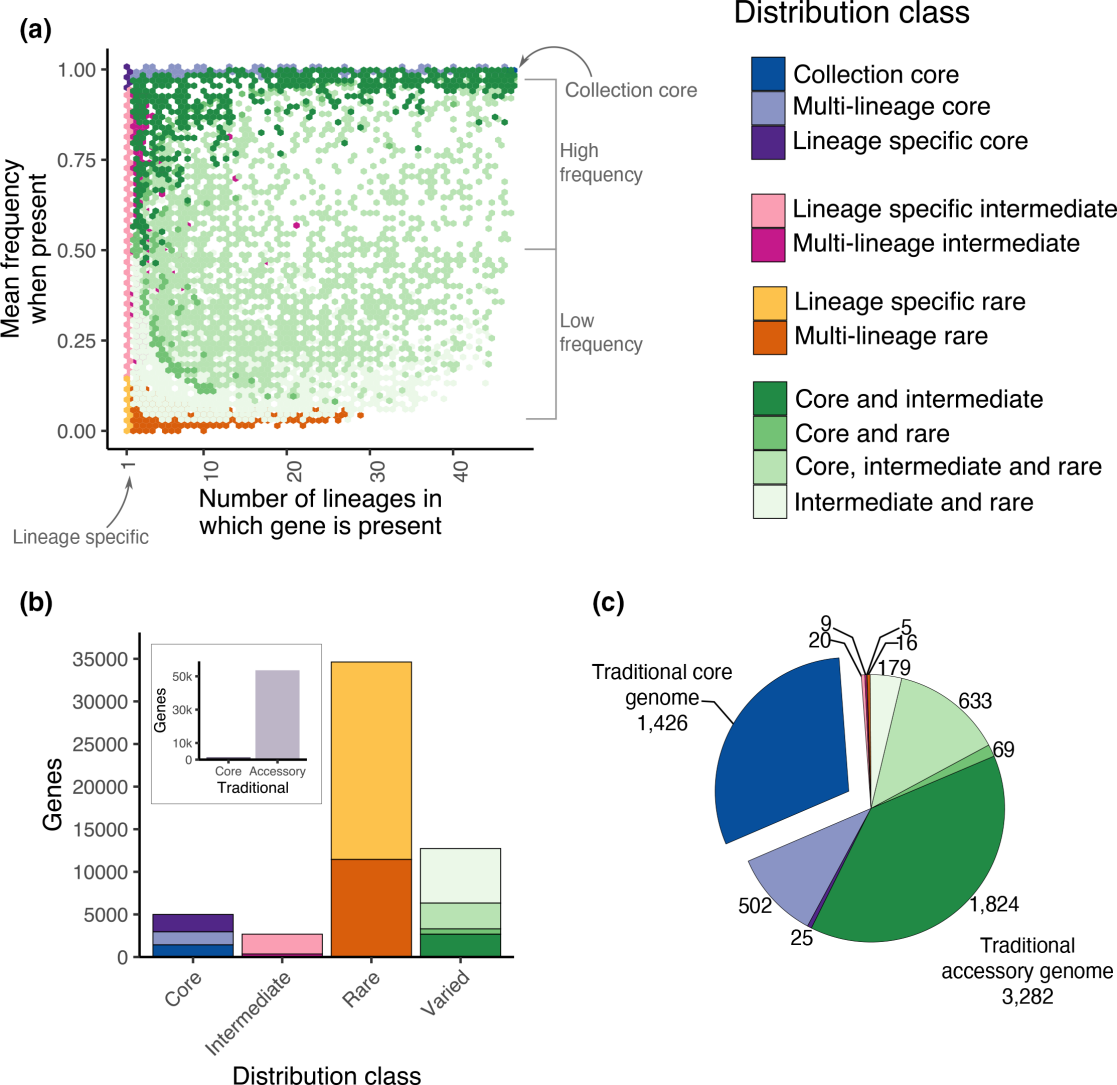
Population-structure aware pan-genome of *E. coli *. (a) Hexagonal binning of all genes of the *E. coli* pan-genome, presented as the number of lineages in which each gene was observed (*x*-axis), against the mean frequency across the lineages containing it (*y*-axis). Each hexagon is coloured by the most common distribution class on the panel (see colour key). The density of points in the figure is present in Fig. S2. (**b)** Number of gene clusters of the *E. coli* pan-genome from each of the novel distribution classes. (**c)** The relative abundance and gene count of each of the distribution classes in a typical *E. coli* genome in the collection. Only the collection core genes represent the traditional set of core genes; the rest represent what would usually all be summarized as the accessory genome.

Fig. 3(a) illustrates the new distribution classes, based on the number of lineages in which they were observed and their mean frequency within those lineages. Only the top right corner represents the traditional set of core genes. The rest of the panel is what is usually summarized as the accessory genome; the colours describe the underlying distribution classes. The plot shows the continuity of gene frequencies across the entire collection, with genes present across almost the entire distribution frequency spectrum; this information is lost by using the traditional binary approach and highlights the increased richness of information that can be obtained and explored using these 13 categories. In the case of *E. coli *, most gene clusters in the pan-genome sit at the extreme ends of the matrix, as described below and in Figs 3(a), S2 and S3.

Within this expanded classification, ‘collection core genes’ are equivalent to the traditional classification of core (assuming a threshold of ≥95 % of the genomes in the collection encoding for a gene for it to be defined as core). In this analysis, the collection core consists of 1426 gene clusters, representing 3 % of the total number of gene clusters comprising the *E. coli* pan-genome (1426/50 039) and 30 % of the total number of genes in a typical *E. coli* genome (defined as the weighted median across the 47 lineages, see Methods, Fig. 3b, c, Table S1).

An additional 1532 gene clusters (3 % of the pan-genome) are now defined as multi-lineage core: that is, they are present in ≥95 % of isolates per lineage in multiple (but not all) lineages (2–46 lineages; Fig. 3b). Another 2040 genes (4 % of all genes) were core to only a single lineage (‘lineage-specific core’; Fig. 3b). Both classes would have been assigned to the accessory genome following the classical definition of the pan-genome, as genes that are core to lineages with low representation in the dataset would have been categorized as rare genes. Importantly, these two additional distribution classes allow us to capture more recent acquisition or loss events that have remained fixed in a respective lineage or lineages.

### The majority of rare and intermediate genes are lineage-specific

The majority of the *E. coli* gene clusters were classified as ‘rare genes’ (Fig. 3b, defined as present in <15 % of isolates of a lineage) in one or multiple lineages within the dataset. In total, 63 % (34 624/55 039) of the *E. coli* pan-genome was classified as rare, with 67 % of all rare genes being specific to a single lineage (23 175/34 624; Fig. 3b). In relation to a single *E. coli* genome, these genes only form 0.1 % of a typical genome (Fig. 3c).

Intermediate frequency gene clusters, by contrast, formed only 4 % (2685/55 039) of the entire gene pool; however, similar to the rare gene clusters, 86 % of intermediate gene clusters (2329/2685) were only observed in a single lineage (‘lineage-specific intermediate’). Rare and intermediate genes observed in multiple lineages were most commonly observed in up to four lineages (‘multi-lineage rare’ and ‘multi-lineage intermediate’, respectively) (Figs 3c and S3). We did not observe any rare or intermediate genes present across more than 30 lineages, and there were no ‘collection rare’ or ‘collection intermediate’ genes in this dataset (Figs 1a, 3a, b and S3).

### A fifth of the pan-genome consists of genes observed in different frequencies across the lineages

‘Varied genes’ were defined as those observed in several lineages, but at different frequencies within the respective lineages. To summarize all of these observations, genes were categorized as ‘core and intermediate’, ‘core, intermediate and rare’, ‘core and rare’, or ‘intermediate and rare’ depending on the combination of frequencies in which they appeared (Fig. 1c). For example, a core and intermediate gene cluster might be core in two lineages, and intermediate in one, whilst a gene core to one lineage but rare in another would be classed as a ‘core and rare’ gene (Fig. 1c). These represented 23 % of the pan-genome (12 732/55 039) (Fig. 3b) and 57 % of all genes in a typical *E. coli* genome (Fig. 3c). In a typical *E. coli* genome, ‘core and intermediate’ genes were commonly observed in more lineages and in higher frequencies within those lineages and represented 38 % of the genes (Figs 3a, c and S3). On the other hand, the group of ‘intermediate and rare’ genes had a lower frequency and were observed in fewer lineages (Figs 3a and S3).

### Low-frequency genes are four times more likely to have been horizontally transferred than high-frequency genes

As the pan-genome in any collection represents a snapshot of the gene pool at the time of sampling, our refined view of the different distribution classes may be used to infer how the genes are gained and lost and can indicate a gene’s future trajectory within a population. For instance, genes that are self-mobile or carried as cargo on mobile genetic elements will have a markedly different pattern of distribution relative to genes that may be in the process of being selectively lost in any particular lineage.

**Fig. 4.**
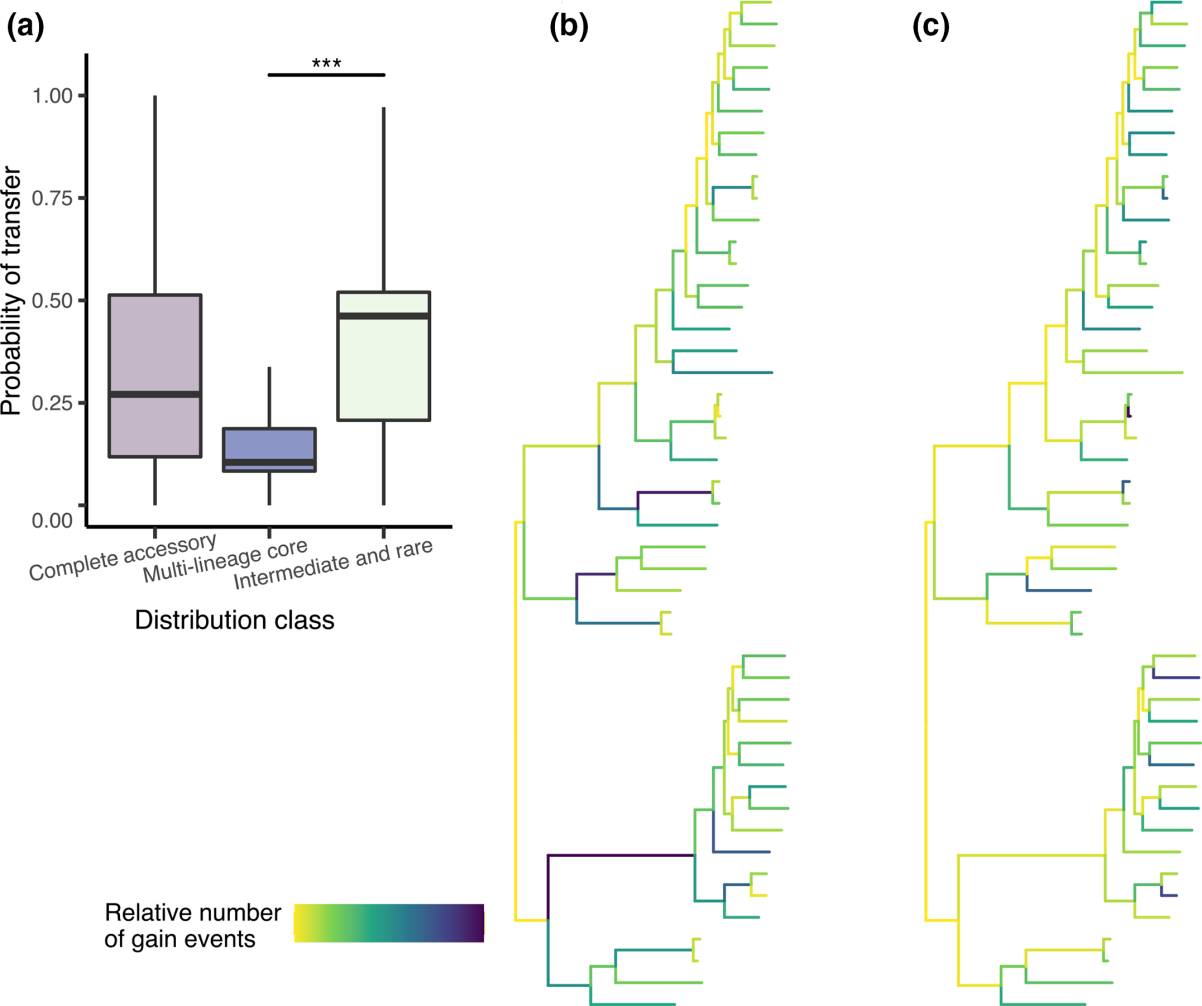
Different evolutionary dynamics of genes within the accessory genome. (a) Inferred probability of transfer using species-tree gene-tree reconciliation for the entire accessory genome (i.e. all 12 distribution classes which make up the accessory genome), only the ‘multi-lineage core’ genes, and only ‘intermediate and rare’ genes (Wilcoxon rank sum test, ***P<0.001). (**b,c)** Number of gain events estimated to have occurred on each branch using ancestral state reconstruction when considering the ‘multi-lineage core’ genes (**b**) or all the ‘intermediate and rare’ genes (**c**). Darker colours indicate that more gain events were estimated to have occurred on a branch.

To assess whether genes from the different distribution classes showed varying evidence of levels of mobility and estimate the probability of genes having been horizontally transferred, we applied a species-tree gene-tree reconciliation method [10] to each gene cluster of the pan-genome. As expected, higher frequency genes (Fig. 3b), i.e. those present in the ‘collection core’, ‘core and intermediate’, and ‘multi-lineage core’ gene sets, were estimated to have the lowest probabilities of having been horizontally transferred (median 0.12, 0.13 and 0.1, respectively) (Figs 4a and S4). Conversely, the lower frequency gene classes, i.e. ‘multi-lineage rare’, ‘multi-lineage intermediate’, ‘intermediate and rare’, and ‘core, intermediate and rare’ gene sets, were estimated to be up to four times more likely to have been horizontally transferred than the high-frequency genes (median probabilities of 0.48, 0.46, 0.44 and 0.31, respectively, Fig. S4). Consistent with this, by counting the total number of gene gain events predicted to have occurred on each branch using ancestral state-reconstruction, multi-lineage core gene gains most commonly occurred along the internal branches (Fig. 4b) whereas ‘intermediate and rare’ genes were predominantly gained at the branch tips (Fig. 4c).

Of the multi-lineage core genes, 54 % could be assigned as basic cellular processes such as metabolism, information storage and processing, and cell signalling (Fig. S5). On the other hand, 73 % of ‘intermediate and rare’ genes were either assigned to a poorly characterized function (often associated with genetic mobility) or of unknown function (Fig. S6).

### Detection of shared horizontally transferred genes between lineages is strongly dependent on unbiased sampling

We observed that the number of ‘intermediate and rare’ genes shared between every two lineages was positively correlated with the size of the two lineages being compared, with larger lineages sharing more mobile genes (Fig. 2a, log linear regression, *R* ^2^=0.45, *P*<2.2e-16). By contrast, we did not observe a relationship between the number of ‘intermediate and rare’ genes shared between every two lineages and their phylogenetic distance (Fig. 2b; linear regression, *R* ^2^=0.005, *P*=0.01). Using our population-structure aware approach to measure sharing of the genes belonging to the different distribution classes suggests a lack of a barrier to gene flow between lineages. That said, our analysis highlights the need to increase sampling of under-studied lineages to overcome sampling-related biases and truly understand the level of horizontal transfer of genes between them.

### Novel distribution classes can highlight lineages with evolutionary trajectories unusual for the species

**Fig. 5.**
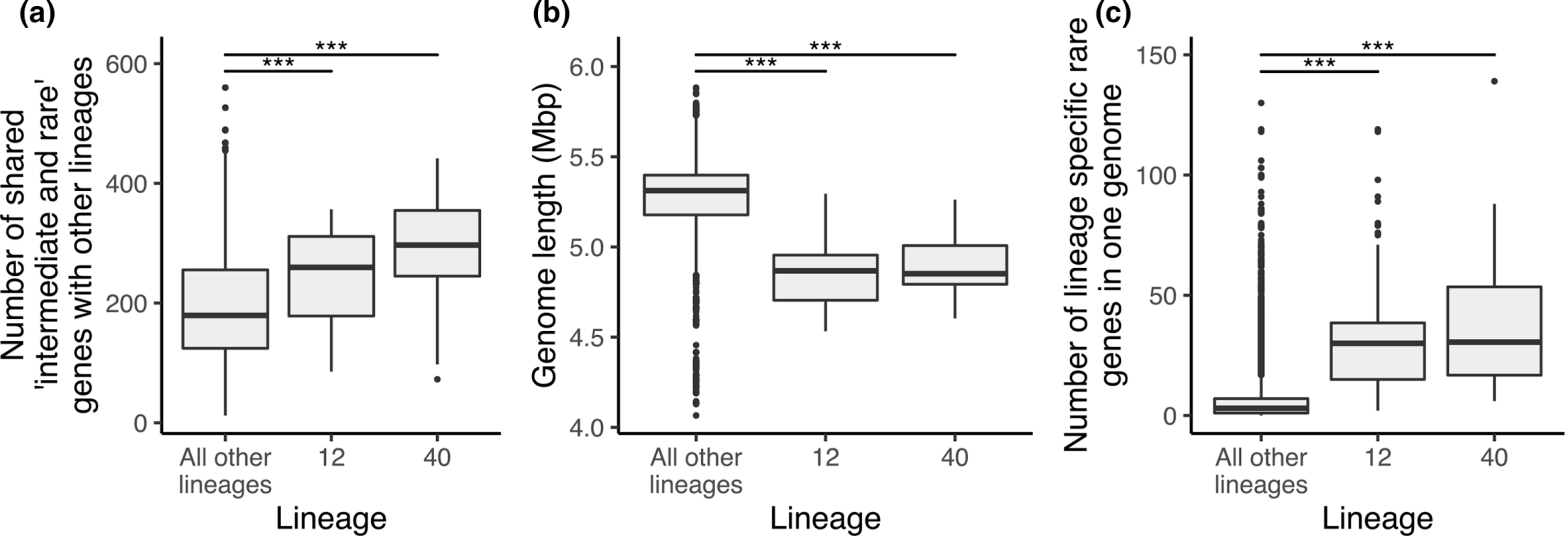
Redefining the pan-genome reveals key insights into particular lineages. (**a)** Number of shared mobile genes per isolate, for isolates belonging to lineage 12, lineage 40 or all other lineages. Counts were corrected for the dependency on the lineage size by measuring gene sharing across repeated subsampling of the lineages, and were corrected for gene sharing driven by phylogenetic relatedness by comparing lineages only from different phylogroups (pairwise Wilcoxon rank sum test, FDR corrected, *P<0.05, **P<0.01, ***P<0.001). (**b)** Genome length of each isolate, for isolates belonging to lineage 12, lineage 40 and all other lineages (pairwise Wilcoxon rank sum test, FDR corrected, *P<0.05, **P<0.01, ***P<0.001).** (c)** Number of ‘lineage specific rare’ genes observed in each isolate, for isolates belonging to lineage 12, lineage 40 and all other lineages (pairwise Wilcoxon rank sum test, FDR corrected, *P<0.05, **P<0.01, ***P<0.001).

We corrected the counts of shared genes due to the bias led by the size of the lineages and any sharing of genes driven by phylogenetic relatedness by repeated subsampling of the lineages (see Methods, Fig. S1). This revealed that two lineages (12 and 40) tended to share more ‘intermediate and rare’ genes than expected compared to other lineages in the collection [pairwise Wilcoxon rank sum test, *P*<0.001, false discovery rate (FDR) corrected, Figs 5a and S7]. Genomes in lineages 12 and 40, however, are smaller than those in other lineages (pairwise Wilcoxon rank sum test, *P*<0.001, FDR corrected, Fig. 5b), and the mean number of lineage-specific rare genes in a single genome was 32 and 30 genes, respectively, compared to five in a typical *E. coli* genome (pairwise Wilcoxon rank sum test, *P*<0.001, FDR corrected; Figs 3c, 5c and S8). Overall, the relative fraction of lineage-specific rare genes in the genomes of these lineages was seven times higher relative to the median fraction in the entire collection (median fraction in collection=0.001; median fraction in lineages 12 and 40: 0.007; Fig. 3c). Similar to the other low-frequency genes, the ‘lineage-specific rare’ genes were also most commonly predicted to be phage-derived or otherwise had other annotations related to genetic mobility (Fig. S5). Exploring these ‘outliers’ can provide insights into adaptive processes used by certain lineages but not others.

## Discussion

To date, the existence of complex population structure and diverse lineages in bacterial populations has not been taken into account in pan-genome analyses. We introduce a population-structure aware classification of the pan-genome as an extended set of 13 classes. Our study reveals distinctive patterns in the evolutionary dynamics of these gene classes, with differences in the relative importance of these gene classes between lineages within *E. coli *. Our approach can be further applied to other bacterial species of public health interest to provide insight into the evolutionary dynamics of genes within such species.

Subcategorizing the genes of the accessory genome allowed us to distinguish the evolutionary dynamics of different gene classes within the accessory genome. Grouping all the genes of the accessory genome together showed a large spread of probabilities of genes being horizontally transferred. Our refined approach showed that low-frequency genes transfer more frequently than high-frequency genes. Importantly, the study of outliers, which disagree with the general trend of each of the distribution classes, can reveal gene-specific evolutionary dynamics, including adaptive processes. For instance, multi-lineage core genes estimated to have high rates of transfer may represent genes that were acquired and fixed independently on multiple occasions and could be cases of convergent evolution and adaptation to similar niches. While many of our observations were already assumed, our more nuanced approach enables the user to elegantly explore these hypotheses and further predict defining properties of lineages.

By expanding the number of distribution classes of the accessory genome relative to traditional approaches, we were able to observe a relationship between the number of rare genes per genome and high levels of sharing of horizontally transferred genes in two lineages, 12 and 40. This relationship has biological implications, as it suggests that the higher levels of gene sharing are driven by an increased ability to gain mobile genes in each genome for isolates belonging to these lineages, or an inability to prevent invasion by foreign selfish elements. In total, 78 % of the isolates from lineage 12 are of sequence type (ST) 10 and 43 % of the isolates in lineage 40 are from ST23. ST10 and ST23 are ubiquitous as they have been described as both commensal and pathogenic, multidrug-resistant, as well as isolated from human and animal sources [23, 24]. These properties have labelled these lineages as generalists and as potential facilitators of gene movement in the population [25]. Here we showed that these differences can be identified and exemplified through more refined analysis of the pan-genome of the entire dataset, as well as within each lineage separately. In doing so, we can also identify lineages that have a greater propensity as vectors for facilitating gene movement. Our observations are just the tip of the iceberg and future studies could further explore the biological differences underpinned by the genetic traits exhibited by different lineages.

It is clear that as available genomic data grow, and our understanding of the population structure becomes richer, a population structure-aware approach to analysing the gene frequency distribution is necessary to overcome several biases inherent in large datasets consisting of variably sampled populations, as these biases can overshadow the true distribution of the genes in a population. For example, using a traditional approach, treating all gene counts across the entire collection equally, genes that are core and specific to a single lineage that has a low representation or penetrance in the collection could be mistaken for rare genes. Identification of these genes is highly important, as being core to only a subset of the population suggests that they have an evolutionary advantage in a particular genetic context or ecological setting [26, 27]. Additionally, genes that are core to a subset of the population are particularly relevant to investigate further for their potential use in diagnostics and epidemiology. Identification of these patterns exemplifies the utility of this approach and its applicability to a wide range of species.

## Supplementary Data

Supplementary material 1

Supplementary material 2
