# Supplementary material for: Different evolutionary trends form the twilight zone of the bacterial pan-genome
Source: Microb Genom. 2021 Sep 24;7(9):000670. doi: 10.1099/mgen.0.000670 (PMC8715436; doi:10.1099/mgen.0.000670)
Supplement: Supplementary material 1 [file mgen-7-0670-s001.pdf]

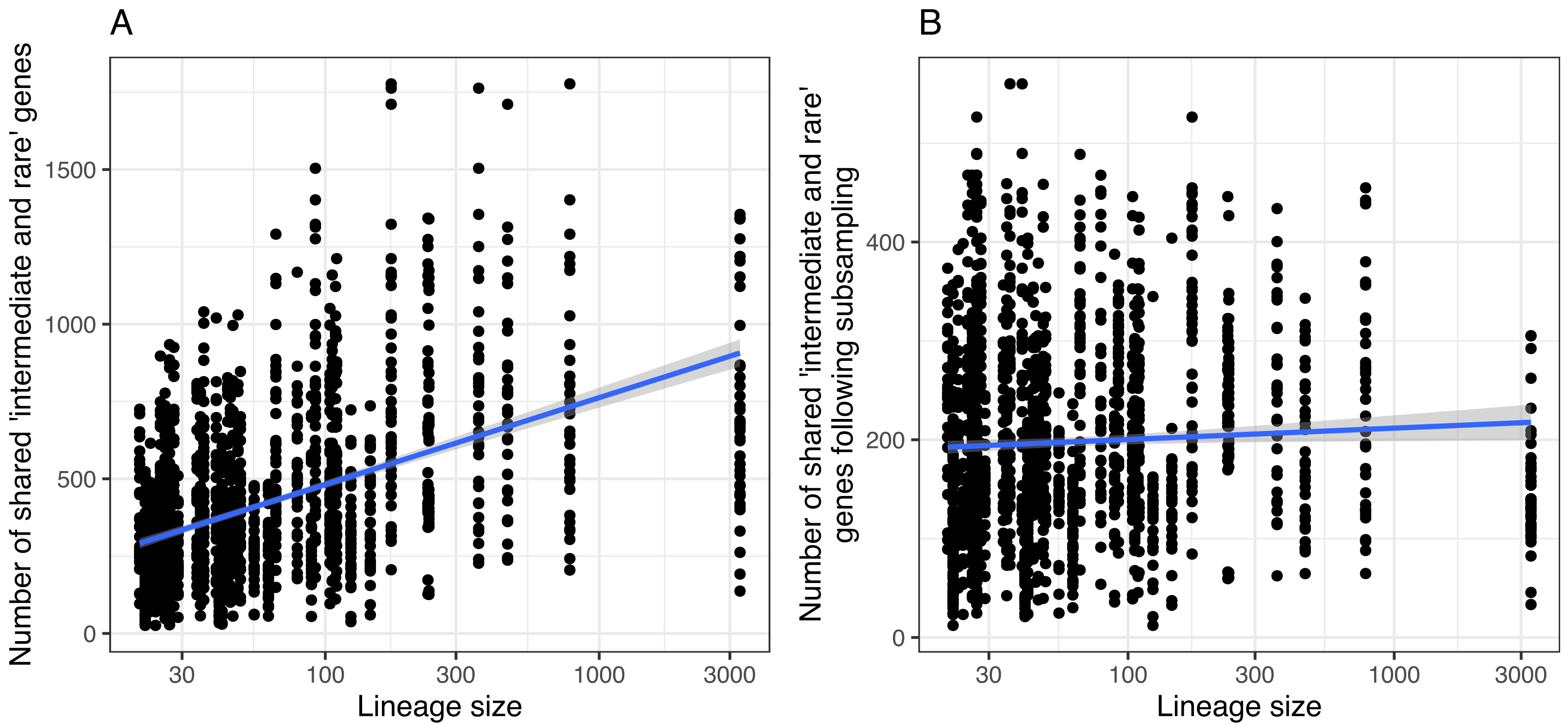

Figure S1: Lineage size correction for number of shared 'intermediate and rare' genes using lineage sub-sampling. A Original number of shared genes betweeen every two lineages, relative to the size of the lineage (log10-transformed). Counts were only considered for lineages belonging to different phylogroups. Line fitted using linear regression, p<2.2e-16, R2=0.22. B Number of shared 'intermediate and rare' genes, corrected by subsampling each lineage to a size of 20 and repeated 40 times. The count presented is mean number of shared 'intermediate and rare' genes across the 40 subsamples, p=0.02, R2=0.

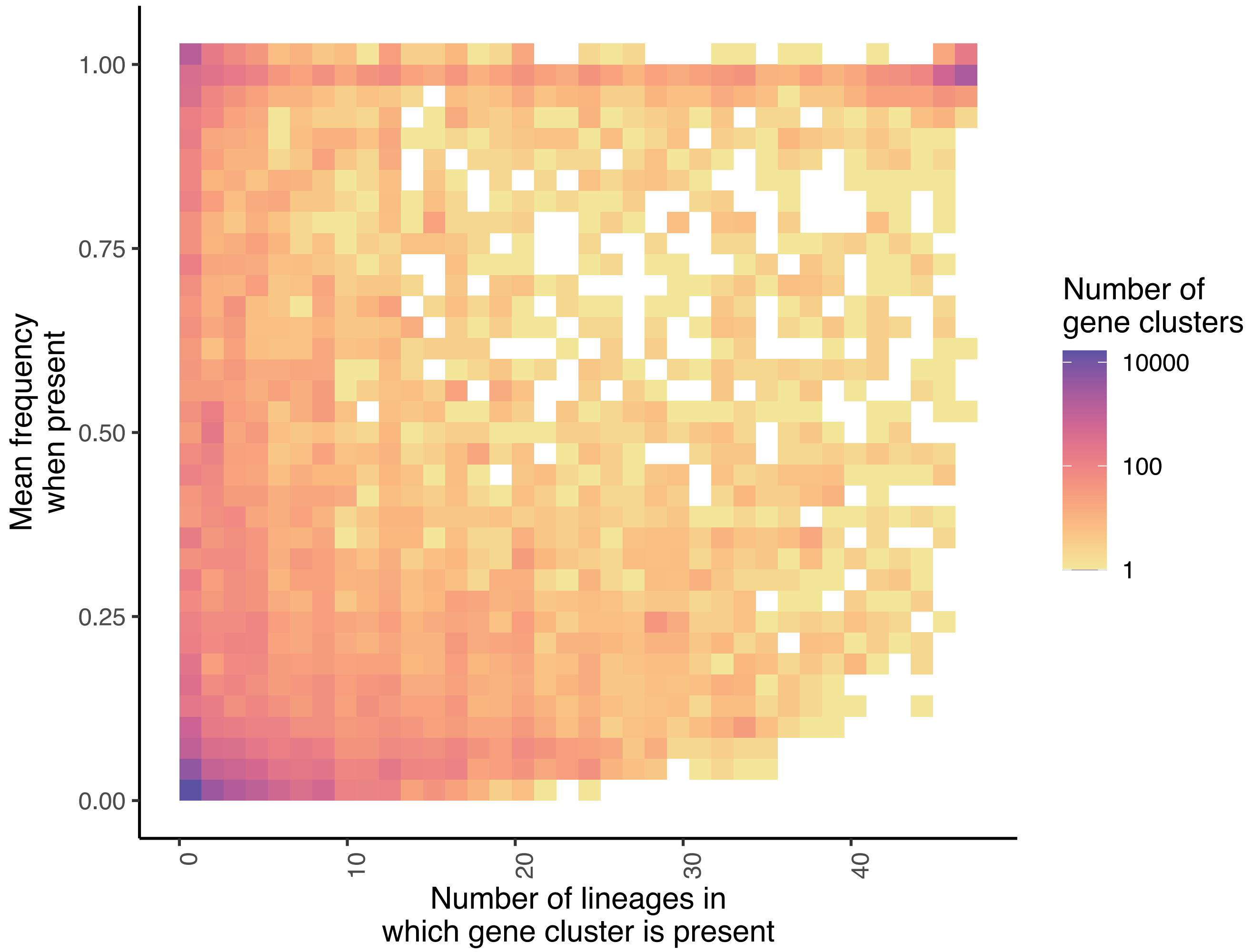

Figure S2: Density of all gene clusters of the *E. coli* pan-genome, presented as the number of lineages in which each gene was observed (x-axis) against the mean frequency across the lineages containing it (y-axis). Each square is coloured by the number of gene clusters with the values, log scaled.

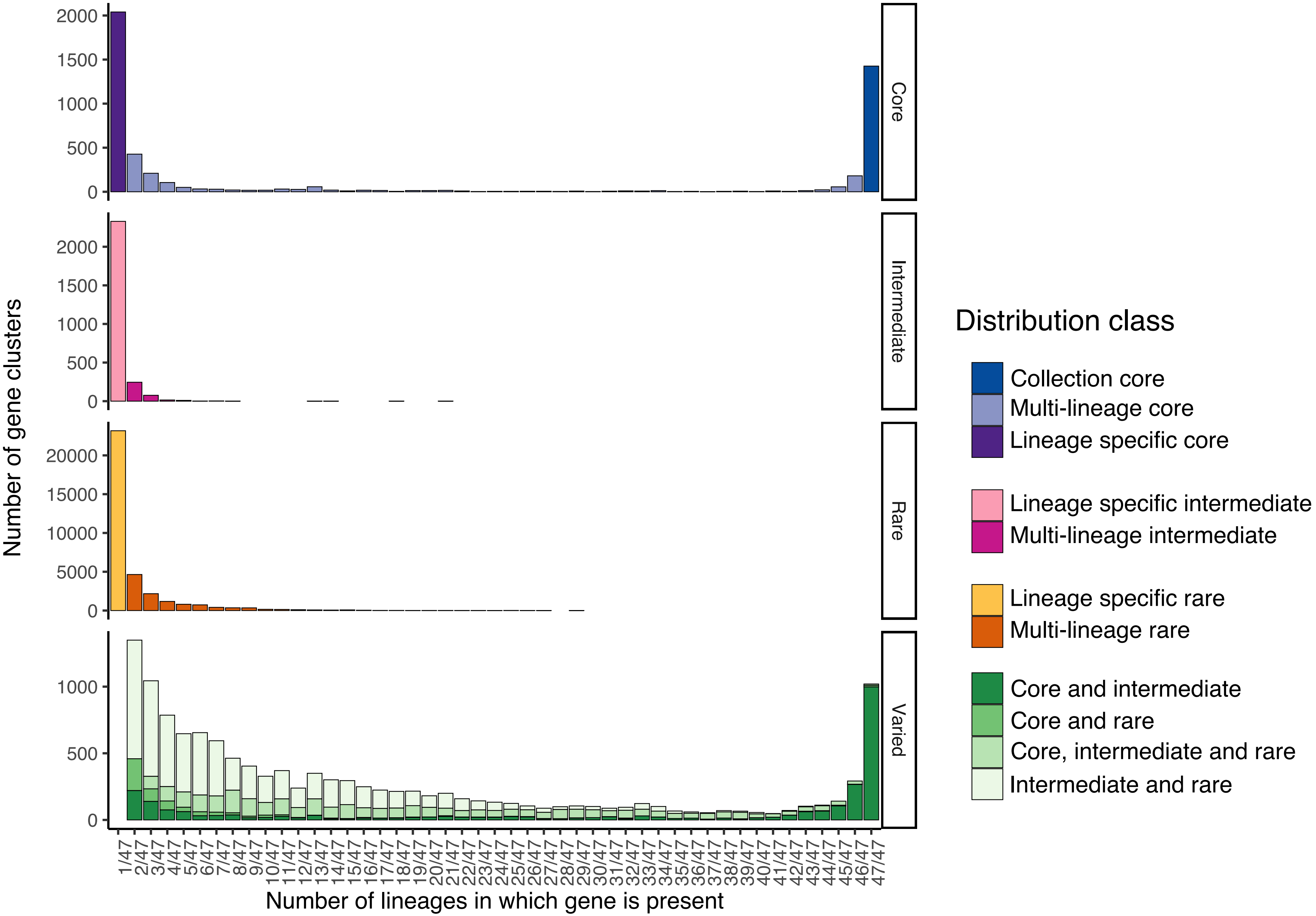

Figure S3: Distribution of the number of genes in each distribution class relative to the number of lineages in which they were found.

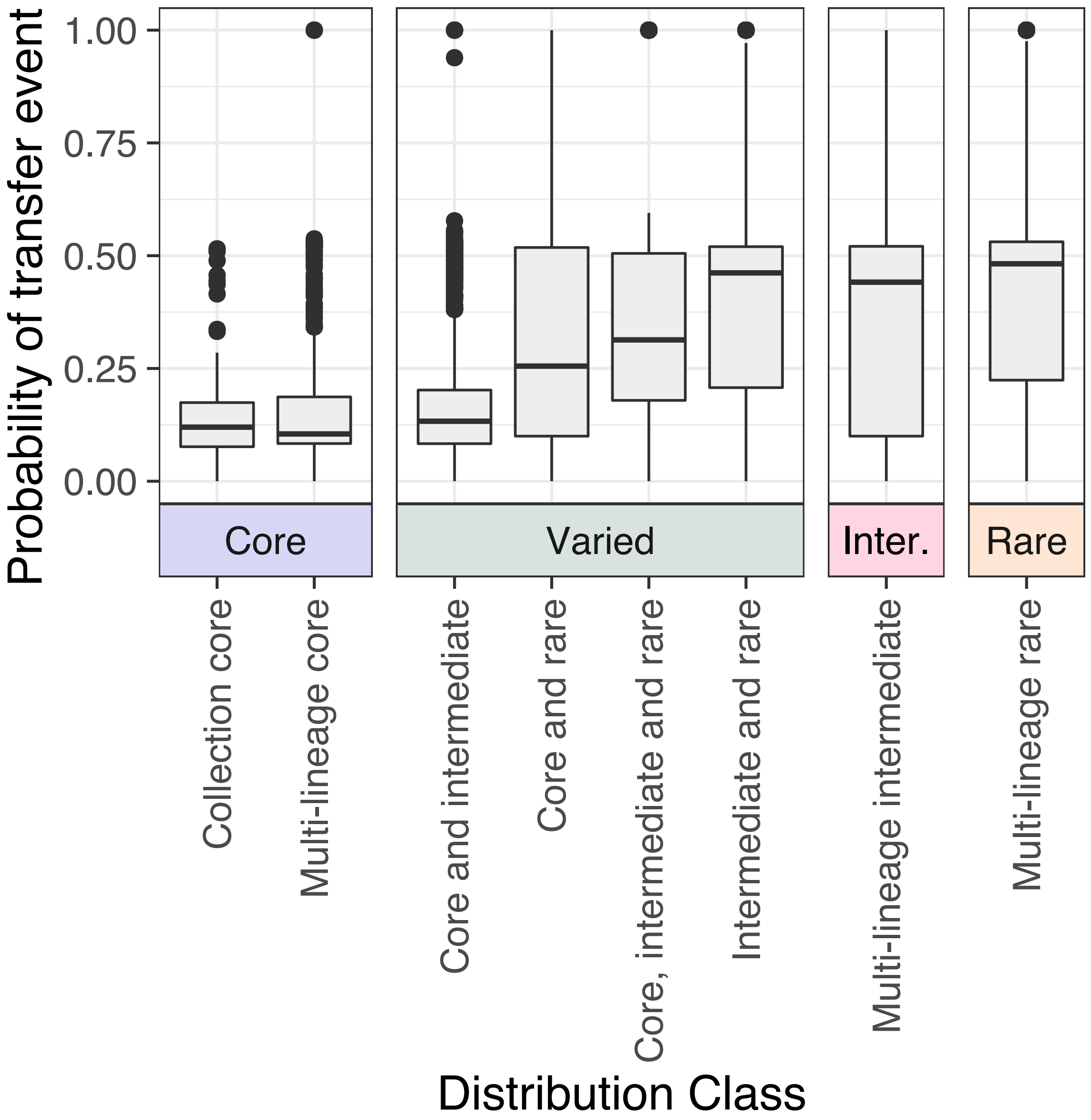

Figure S4: Inferred probability of transfer using gene tree/ species tree reconciliation for the genes belonging to the different distribution classes.

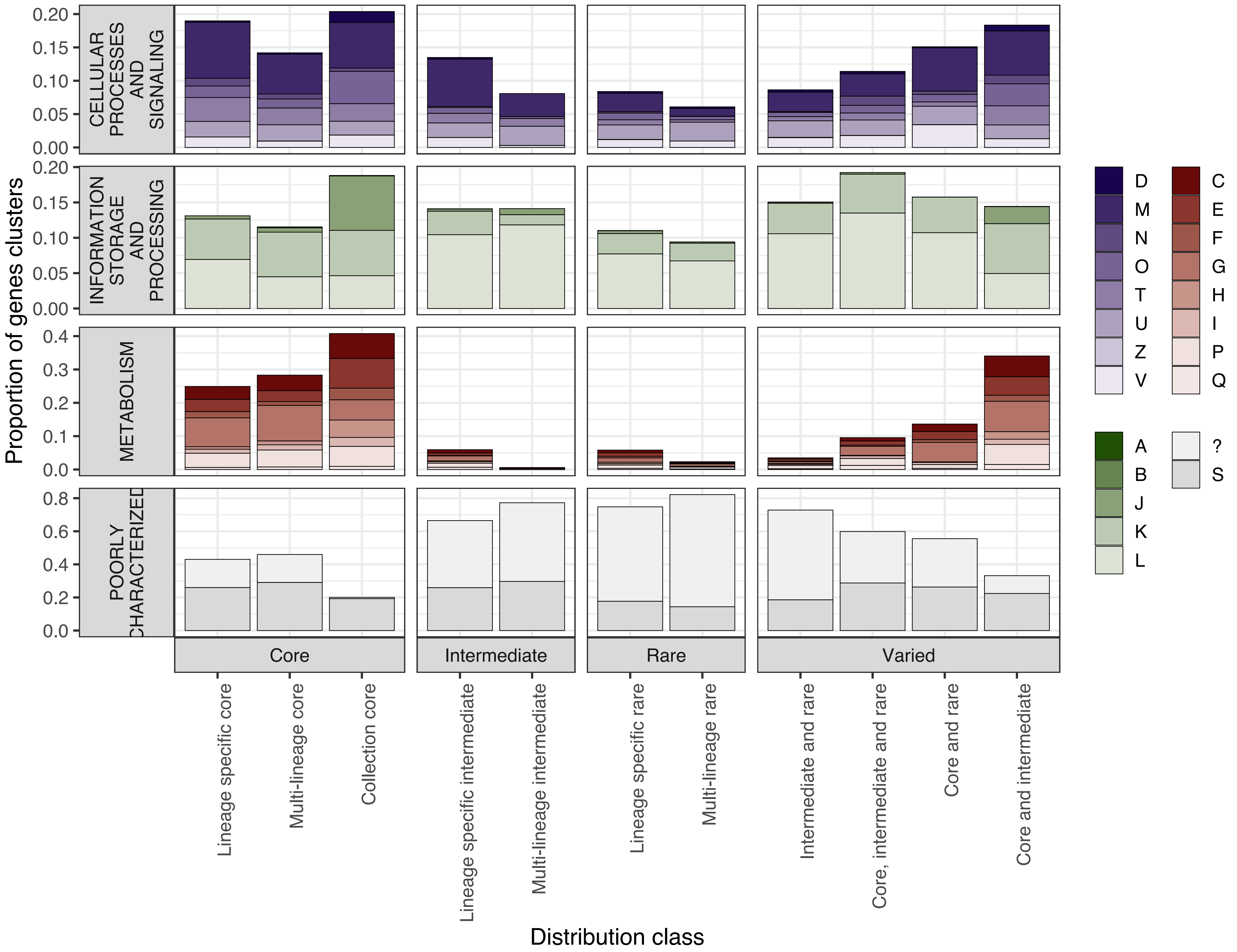

Figure S5: Fraction of genes from each occurrence class which were assigned each of the COG categories. D (Cell cycle control, cell division, chromosome partitioning), M (Cell wall/membrane/envelope biogenesis), N (Cell motility), O (Post-translational modification, protein turnover, and chaperones), T (Signal transduction mechanisms), U (Intracellular trafficking, secretion, and vesicular transport), Z (Cytoskeleton), V (Defence mechanisms), A (RNA processing and modification), B (Chromatin structure and dynamics), J (Translation, ribosomal structure and biogenesis), K (Transcription), L (Replication, recombination and repair), C (Energy production and conversion), E (Amino acid transport and metabolism), F (Nucleotide transport and metabolism), G (Carbohydrate transport and metabolism), H (Coenzyme transport and metabolism), I (Lipid transport and metabolism), P (Inorganic ion transport and metabolism), Q (Secondary metabolites biosynthesis, transport, and catabolism), S (Function unknown) and “?” (unassigned).

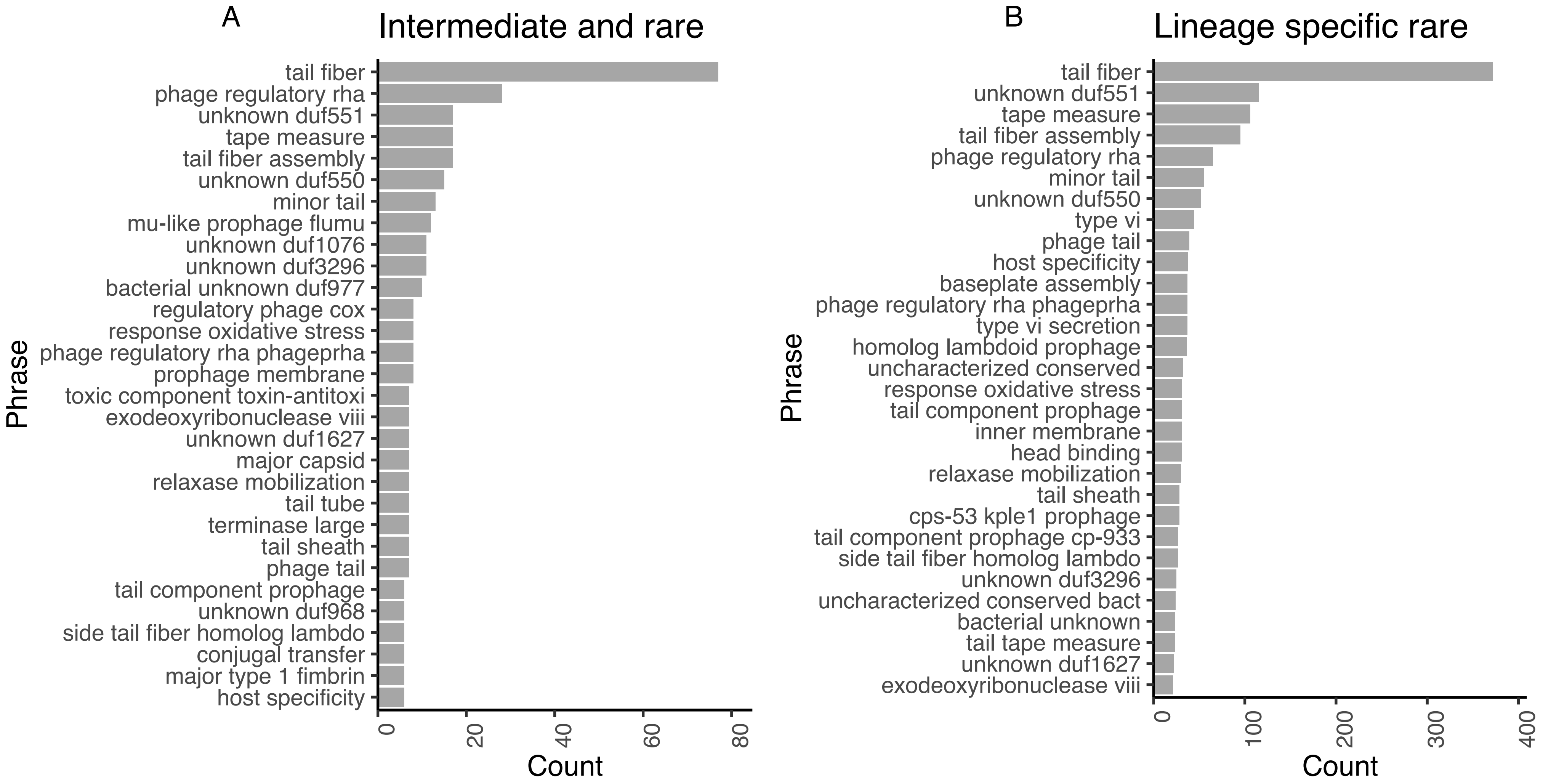

Figure S6: Top thirty predicted phrases for genes belonging to the "Poorly Characterised" COG category, for genes belonging to two low frequency distribution classes: 'intermediate and rare' genes (A) and 'lineage-specific rare' genes (B). The majority of the genes in these distribution classes were assigned a COG category of poorly characterised (Supplementary Figure S5).

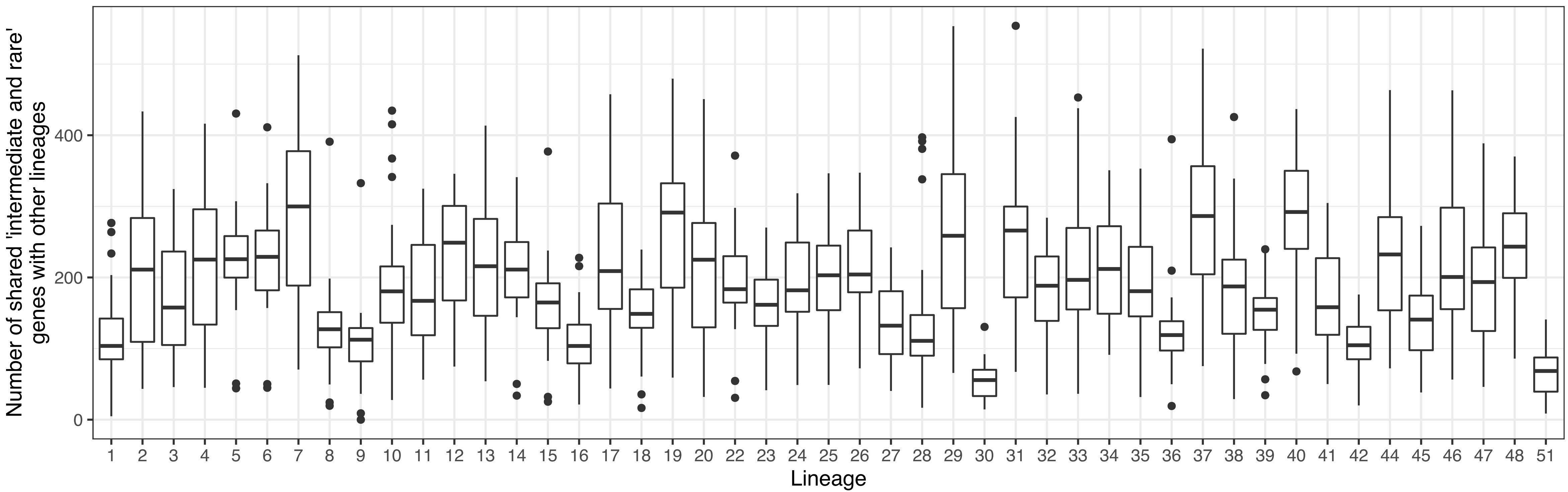

Figure S7: Number of shared intermediate and rare genes per isolate, for all lineages. Counts were only considered between lineages belonging to different phylogroups, and were calculated on a repeated subsampling of all lineage to correct for lineage size (Figure S1).

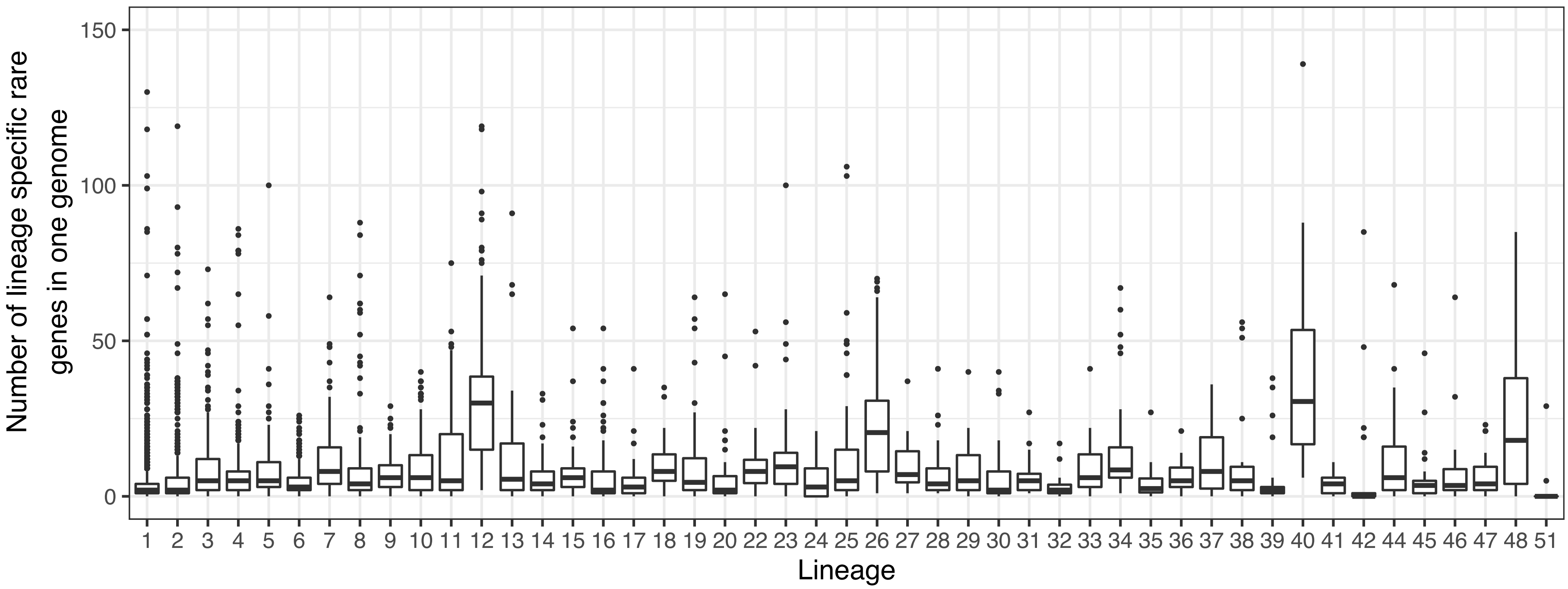

Figure S8: Number of ‘lineage specific rare’ genes observed in each isolate, for isolates belonging to each of the lineages
